# Renin-angiotensin blocker use is associated with improved cardiovascular mortality in Indian patients with mild-moderate chronic kidney disease—findings from the ICKD study

**DOI:** 10.3389/fmed.2022.1060148

**Published:** 2022-12-20

**Authors:** Narayan Prasad, Ashok Kumar Yadav, Monica Kundu, Ajay Jaryal, Dipankar Sircar, Gopesh Modi, Manisha Sahay, Natarajan Gopalakrishnan, Sanjay Vikrant, Santosh Varughese, Seema Baid-Agrawal, Shivendra Singh, Sishir Gang, Sreejith Parameswaran, Arpita Ghosh, Vivek Kumar, Vivekanand Jha

**Affiliations:** ^1^Department of Nephrology, Sanjay Gandhi Postgraduate Institute of Medical Science, Lucknow, India; ^2^Department of Experimental Medicine and Biotechnology, Postgraduate Institute of Medical, Chandigarh, India; ^3^George Institute for Global Health India, Delhi, India; ^4^Department of Nephrology, Indira Gandhi Medical College, Shimla, India; ^5^Department of Nephrology, Institute of Post Graduate Medical Education and Research, Kolkata, India; ^6^Department of Nephrology, Samarpan Kidney Institute and Research Center, Bhopal, India; ^7^Osmania Medical College, Osmania General Hospital, Hyderabad, India; ^8^Department of Nephrology, Rajiv Gandhi Government General Hospital, Chennai, India; ^9^Department of Nephrology, Christian Medical College, Vellore, India; ^10^Department of Nephrology and Transplant Center, Sahlgrenska University Hospital, University of Gothenburg, Gothenburg, Sweden; ^11^Department of Nephrology Institute of Medical Science, Banaras Hindu University, Varanasi, India; ^12^Department of Nephrology, Muljibhai Patel Urological Hospital, Nadiad, India; ^13^Department of Nephrology, Jawaharlal Institute of Postgraduate Medical Education and Research, Pondicherry, India; ^14^Department of Nephrology, Postgraduate Institute of Medical Education and Research, Chandigarh, India; ^15^School of Public Health, Imperial College, London, United Kingdom; ^16^Prasanna school of Public Health, Manipal Academy of Higher Education, Manipal, India

**Keywords:** angiotensin-converting enzyme inhibitors, angiotensin receptor blockers, chronic kidney disease, cardiovascular mortality, all-cause mortality

## Abstract

**Introduction:**

Angiotensin-converting enzyme inhibitors (ACEI) and angiotensin receptor blockers (ARB) are the antihypertensive drug class of choice in patients with chronic kidney disease (CKD). Head-to-head comparisons of the renal or non-renal outcomes between ACEI/ARB users and nonusers have not been conducted in all population groups. We examined the renal and cardiovascular outcomes in users and nonusers enrolled in the Indian Chronic Kidney Disease (ICKD) Study.

**Methods:**

A total of 4,056 patients with mild-moderate CKD were studied. Patients were categorized as ACEI/ARB users or nonusers. Major adverse kidney events [ESKD (end stage kidney disease), ≥50% decline in eGFR and kidney death], all-cause mortality, and cardiovascular mortality were analyzed over a median follow-up period of 2.64 (1.40, 3.89) years between the two groups.

**Results:**

Out of a total of 4,056 patients, 3,487 (87%) were hypertensive. The adjusted sub-hazard ratio (SHR) and 95 % CI for ACEI /ARB users was 0.85 (0.71, 1.02) for MAKE, 0.80 (0.64, 0.99) for a 50% decline in eGFR, and 0.72 (0.58, 0.90) for ESKD. For cardiovascular mortality, ACEI/ARB users were at lower risk (SHR = 0.55, 95% CI: 0.34, 0.88). Diuretic users were at increased risk of all-cause mortality (HR = 1.95, 95% CI: 1.50, 2.53) and cardiovascular mortality (adjusted SHR = 1.73, 95% CI: 1.09, 2.73). There was non-significant association between the use of other antihypertensives and any of the end points.

**Discussion:**

ACEI/ARB use is associated with slower rate of decline in eGFR in those with CKD stage 1-3. ACEI/ARB users had a significantly lower risk of renal outcomes, and cardiovascular mortality.

## Introduction

Hypertension is both a consequence and risk factor for the development and progression of chronic kidney disease (CKD). Optimal blood pressure control in patients with CKD usually requires the use of multiple anti-hypertensive agents (1, 2). Drugs that interrupt the renin-angiotensin-aldosterone system (RAAS) are recommended as the preferred antihypertensives because of their additional salutary effects on the progression of CKD (3, 4) and the development of cardiovascular disease (CVD), a major cause of morbidity and mortality in this population (5–7). These agents may have differential effects in those with and without albuminuria (8). Other antihypertensives are also widely used in patients with CKD (9, 10). Calcium channel blockers (CCBs), and beta-blockers (BBs) may be associated with an increased risk of cardiovascular outcomes or death in the CKD population (11, 12). Head-to-head comparisons of the renal or nonrenal outcomes amongst users of different antihypertensive drug classes in CKD are not available in all populations (7, 11). In particular, data from developing world are scarce. A recent study on the prescription patterns revealed that <50% of patients with CKD stage 3/4 in India were receiving angiotensin pathway blockers (2).

In this manuscript, we describe the antihypertensive drug usage in a wellcharacterized cohort of patients with mild-moderate CKD enrolled in the Indian Chronic Kidney Disease (ICKD) Study, and examine the impact of angiotensin pathway blockers on the progression of CKD as well as all-cause mortality and cardiovascular mortality.

## Materials and methods

### Study population

The details of the ICKD cohort study design have already been published (13). Briefly, the study is recruiting adult participants with mild to moderate CKD; estimated glomerular filtration rate (eGFR) between 15 and 60 ml/min/1.73 m^2^ or proteinuria >500 mg/d, from 11 large hospitals in India. Those with eGFR <15 ml/min/1.73 m^2^, kidney transplant recipients, and those on immunosuppressive drugs, with malignancy or poor functional status are excluded. The study has approval from the institutional review board at each center, and all participants provide written informed consent.

Demographic details, diagnosis, comorbidities, clinical, laboratory, and treatment details are recorded and stored anonymously in a secure central database. All the enrolled patients are followed regularly, and outcome events are recorded.

### CKD staging and study variable definition

Participants were categorized into different CKD stages as per Kidney Disease Improving Global Outcomes (KDIGO) criteria (14). GFR is estimated using the 2012 Chronic Kidney Disease Epidemiology Collaboration Equation with serum creatinine measured using assays traceable to isotope dilution mass spectrometry (IDMS) reference. Albuminuria is defined by dipstick positivity, albumin-creatinine ratio, or 24-h quantification. Educational status is categorized as per prevalent educational tiers. Economic status is categorized into quartiles. Rural residents were defined as participants residing in villages, and urban residents are those living in areas designated as towns and cities. Hazardous occupational exposure is defined as regular exposure to sand, dust, or chemicals, or working barefoot in fields. Alternative medication use is defined as the use of indigenous, ayurvedic, or other unregulated medications.

Diabetes is defined as fasting plasma glucose of >126 mg/dl, glycated hemoglobin of ≥6.5%, or the use of glucose-lowering drugs. Hypertension is defined as blood pressure >140/90 mm Hg or the use of antihypertensive therapy. CVD is identified by a history of heart failure, coronary artery disease, prior revascularization, stroke, or peripheral vascular disease. All comorbidities are either self-reported or on the basis of chart review. Body mass index (BMI) is categorized as underweight (<18 kg/m^2^), normal (18–24 kg/m^2^), and overweight (≥25 kg/m^2^).

### Use of antihypertensive agents in different stages of CKD

All prescriptions were captured from records. We were primarily interested in the use of ACEI or ARBs, CCBs, BBs, and diuretics at the point of entry to the study. Use of these agents was confirmed by reviewing the prescription of the patient, which is entered into the ICKD database. Patients were categorized as ACEI/ARB user at baseline if they had been on either of these agents for >3 months. We also analyzed the ACEI or ARB use in albuminuric and non-albuminuric subjects. In analysis of factors associated with the use of ACEI/ARB (coded as a binary variable), we included CKD stage, age at enrolment (≥60 vs. <60 years), sex, annual household income, residence, education level, diabetes mellitus, obesity (body mass index ≥25 kg/m^2^), uncontrolled systolic (≥140 mm Hg) and diastolic (≥90 mm Hg) blood pressures, albuminuria, concurrent use of other antihypertensive medications namely (CCBs and BBs), diuretic and statins.

Further, to examine ACEI/ARB use for each of the CKD stage in the entire cohort, usage and non-usage of ACEI/ARB was classified on the basis of entry into each stage of CKD and was analyzed in separate multivariable logistic models.

### Prediction of risk of developing adverse outcomes based on use of antihypertensive agents

We used unadjusted and adjusted Cox models to examine the risk major adverse kidney events (MAKE) (defined as a composite of ESKD, ≥50% decline in eGFR and kidney death), ≥50% GFR decline, ESKD, all-cause mortality and CVD mortality, among users of each antihypertensive drug class adjusting for the baseline covariates including age, residence, sex, income, presence or absence of heart failure, eGFR, albuminuria, diabetes mellitus, obesity, blood pressure, aspirin use, statin use, and a family history of stroke. Deaths were identified through retrieval of death certificates, review of hospital records or reports from next of kin.

### Statistical analysis

Descriptive statistics were used to describe the demographic and clinical characteristics and prescription use patterns of study subjects. Continuous data were presented as mean ± standard deviation (SD) or median (25th, 75th percentile), and categorical data were presented as frequency (percentage). For the purpose of analysis, complete case data were considered. Unadjusted and adjusted logistic regression models were used to assess the association between ACEI/ARB users and nonusers and baseline demographic and clinical characteristics with prescription use as an outcome of interest. Cox proportional hazard models were used to study the association of time to occurrence of events with reference to different anti-hypertensive drugs and after adjusting for other covariates including baseline age, residence, sex, income, heart failure, eGFR, baseline albuminuria, diabetes, obesity, blood pressure, aspirin use, statin use, and family history of stroke. We used the Cox proportional hazard model to estimate the hazard ratio (HR), to capture the effect of an intervention on an outcome of interest over the time as compared to the control group. We used competing risk model to estimate the sub-hazard ratio (SHR) accommodating the risk of an event whose occurrence could preclude the occurrence of the primary outcome of interest. For CVD mortality as an outcome of interest, death due to any other cause was considered as competing event. For 50% GFR decline, ESRD and MAKE as outcomes of interest, non-renal death was considered as competing event. *P* value <0.05 is considered to be significant.

## Results

### Baseline characteristics

The baseline sociodemographic and clinical characteristics of the overall study cohort as well as users and nonusers of ACEIs/ARBs are shown in Tables 1, 2. Mean age of the patients were 50 years; a majority (67%) were males and from rural background (66%). Around 27% were illiterate, 18.6% were current tobacco users, and 87, 37.5, and 21.8% had hypertension, diabetes and CVD respectively. About 44% had BMI of more than 25 kg/m^2^, i.e., were overweight or obese. A total of 1,849 (46.6%) participants were using ACEIs/ARBs, followed by CCBs (1,688, 42.6%), diuretics (1,137, 28.7%) and BBs (1,076, 27.1%). A total of 1,602 (40.4%) participants were receiving statins. Use of ACEIs/ARBs at baseline in albuminuric patients was significantly more as compared to non-albuminuric patients (32 vs. 20%, *p* < 0.01).

**Table 1:** Baseline sociodemographic characteristics of participants in the study.

**Characteristics**	**Total (*N* = 4,056)**	**ACEI/ARB users (*N* = 1,849)**	**ACEI/ARB nonusers (*N* = 2,117)**	**Missing**
Age (years)	50.3 ± 11.8	49.4 ± 11.6	51.4 ± 11.7	0 (0)
**Sex**				
Female	1,331 (32.8)	611 (33.0)	684 (32.3)	0 (0)
Male	2,725 (67.2)	1,238 (66.9)	1,433 (67.7)	
Duration of kidney disease (months)	38.3 ± 53.0	45.1 ± 58.8	32.5 ± 46.8	32 (0.8)
BMI (kg/m^2^)	24.8 ± 4.8	25.4 ± 4.8	24.3 ± 4.7	103 (2.5)
**BMI**				
≥25	1,741 (44.0)	899 (49.7)	813 (39.5)	103 (2.5)
18–24	1,988 (50.3)	842 (46.6)	1,103 (53.6)	
<18	224 (5.7)	68 (3.8)	143 (6.9)	
Waist/hip ratio	1.05 (1.02, 1.09)	1.06 (1.02, 1.1)	1.04 (1.01, 1.08)	964 (23.8)
**Place of residence**				
Rural	2,626 (66.0)	1,150 (63.1)	1,411 (68.4)	80 (2.0)
Urban	1,350 (34.0)	674 (36.9)	652 (31.6)	
**Education level**				
Illiterate	1,088 (26.9)	425 (23.1)	636 (30.2)	18 (0.4)
Below high school	1,374 (34.0)	638 (34.6)	707 (33.6)	
Completed school	538 (13.3)	275 (14.9)	249 (11.8)	
College and above	1,038 (25.7)	505 (27.4)	514 (24.4)	
Hazardous occupational exposure	2,035 (50.4)	904 (49.1)	1,087 (51.6)	18 (0.4)
Current tobacco user	747 (18.6)	322 (17.6)	415 (19.8)	43 (1.1)
Current alcohol user	301 (7.5)	139 (7.6)	161 (7.7)	43 (1.1)
Physically active	1,718 (42.8)	869 (47.5)	827 (39.5)	43 (1.1)
Non-vegetarian diet	2,601 (65.3)	1,254 (68.6)	1,308 (63.2)	75 (1.9)
Access to piped water supply	1,975 (48.9)	889 (48.2)	1,022 (48.5)	18 (0.4)
Annual household income (USD)	1,680 (1,008, 4,200)	1,848 (1,008, 5,040)	1,680 (1,008, 4,200)	41 (1.0)
Annual household medical expenditure (USD)	286 (84, 571)	285.6 (84, 537.6)	285.6 (84, 588)	0 (0.0)
Medical insurance	1,276 (32.1)	645 (35.4)	610 (29.5)	77 (1.9)
Incurred out of pocket medical expenditure	3,352 (83.0)	1,476 (80.1)	1,797 (85.3)	18 (0.4)
Missed hospital visit/drugs due to financial constraints	428 (10.6)	192 (10.4)	212 (10.1)	18 (0.4)

Data presented as mean ± standard deviation, median (25th, 75th percentile) or number (percentage).

BMI, body mass index; USD, United States Dollar.

**Table 2:** Baseline clinical characteristics of participants in the study.

**Characteristics**	**Total (*N* = 4,056)**	**ACEI/ARB users (*N* = 1,849)**	**ACEI/ARB nonusers (*N* = 2,117)**	**Missing**
Hypertension	3,487 (87.0)	1,849 (100)	1,586 (75.3)	49 (1.2)
Diabetes	1,485 (37.5)	820 (44.5)	647 (30.8)	96 (2.4)
CVD	876 (21.8)	425 (23.1)	435 (20.7)	33 (0.8)
Renal stone disease	474 (11.8)	217 (11.8)	244 (11.6)	27 (0.7)
Recurrent UTI	442 (10.9)	199 (10.8)	237 (11.3)	27 (0.7)
Alternative drug use	923 (22.9)	447 (24.2)	449 (21.3)	16 (0.4)
NSAID use	626 (15.6)	319 (17.4)	296 (14.1)	43 (1.1)
History of AKI	268 (6.7)	110 (6.01)	146 (6.9)	43 (1.1)
Required dialysis	231 (5.8)	118 (6.5)	107 (5.1)	43 (1.1)
Underwent renal biopsy	686 (17.1)	441 (24.1)	220 (10.5)	43(1.1)
SPB ≥ 140 mmHg	1,555 (39.3)	767 (42.5)	749 (36.2)	101 (2.5)
DBP ≥ 90 mmHg	1,297 (33.1)	606 (33.9)	657 (32.0)	133 (3.3)
eGFR (ml/min/1.73m^2^)	44.1 (16.1)	47.0 (17.4)	41.7 (14.1)	0
Hemoglobin (g/dl)	11.9 (1.9)	12.1 (1.9)	11.8 (1.9)	143 (3.5)
**Anemia** ^*^				
Mild	1,305 (33.4)	566 (51.5)	718 (51.9)	143 (3.5)
Moderate	1,176 (30.1)	518 (47.1)	626 (45.3)	
Severe	58 (1.5)	16 (1.5)	37 (2.7)	
Serum creatinine (mg/dl)	1.7 (1.5, 2.0)	1.7 (1.4, 1.9)	1.8 (1.5, 2.1)	0
Serum calcium (mg/dl)	9 (8.5, 9.4)	9 (8.5, 9.4)	9 (8.5, 9.5)	290 (7.2)
Serum inorganic phosphorus (mg/dl)	4.0 (3.3, 4.5)	4 (3.3, 4.5)	3.9 (3.3, 4.4)	340 (8.4)
Serum albumin (g/dl)	4.0 (3.5, 4.4)	3.9 (3.5, 4.4)	4 (3.5, 4.3)	225 (5.6)
Serum uric acid (mg/dl)	6.4 (5.2, 7.6)	6.4 (5.2, 7.6)	6.4 (5.3, 7.6)	842 (20.8)
Total cholesterol (mg/dl)	166 (133, 200)	162 (129.6, 199.2)	170 (136.3, 200)	1,538 (37.9)
Triglycerides (mg/dl)	138 (110, 177)	139 (110, 178.5)	136 (109, 175)	1,647 (40.6)
**Urine albumin creatinine ratio (mg/g)**				
<300	2,817 (74.5)	1,199 (68.2)	1,558 (79.9)	272 (6.7)
300–1,000	583 (15.4)	309 (17.6)	264 (13.5)	
>1,000	384 (10.1)	251 (14.3)	129 (6.6)	
Statin use	1,602 (40.4)	996 (53.9)	606 (28.6)	90 (2.2)
Beta-Blocker use	1,076 (27.1)	443 (23.9)	633 (29.9)	90 (2.2)
Calcium-channel blocker use	1,688 (42.6)	751 (40.6)	937 (44.3)	90 (2.2)

Data presented as mean ± standard deviation, median (25th, 75th percentile) or number (percentage).

eGFR, estimated glomerular filtration rate; ACR, albumin creatinine ratio; ACEI, angiotensin converting enzyme inhibitor; ARB, angiotensin receptor blocker; SPB, systolic blood pressure; DBP, diastolic blood pressure.

* Anemia was classified as per WHO criteria: mild anemia-Hb; 11.0–11.9 mg/dl for females and 11–12.9 mg/dl for males, Moderate anemia—Hb; 8.0–10.9 mg/dl for females and males and Severe anemia- Hb < 8 mg/dl for females and males.

The majority of the patients were in CKD stage G3 (78.65%) (Supplementary Table 1). Males outnumbered females across all stages of CKD. The proportion of participants on ACEIs/ARBs was 72% in stage 1, 58.9% in stage 2, 46.8% in stage 3, and 30.5% in stage 4 of CKD. The use of diuretics, BBs and CCBs increased with advancing CKD stages (Supplementary Table 1).

### Outcome events

**Figure 1:**
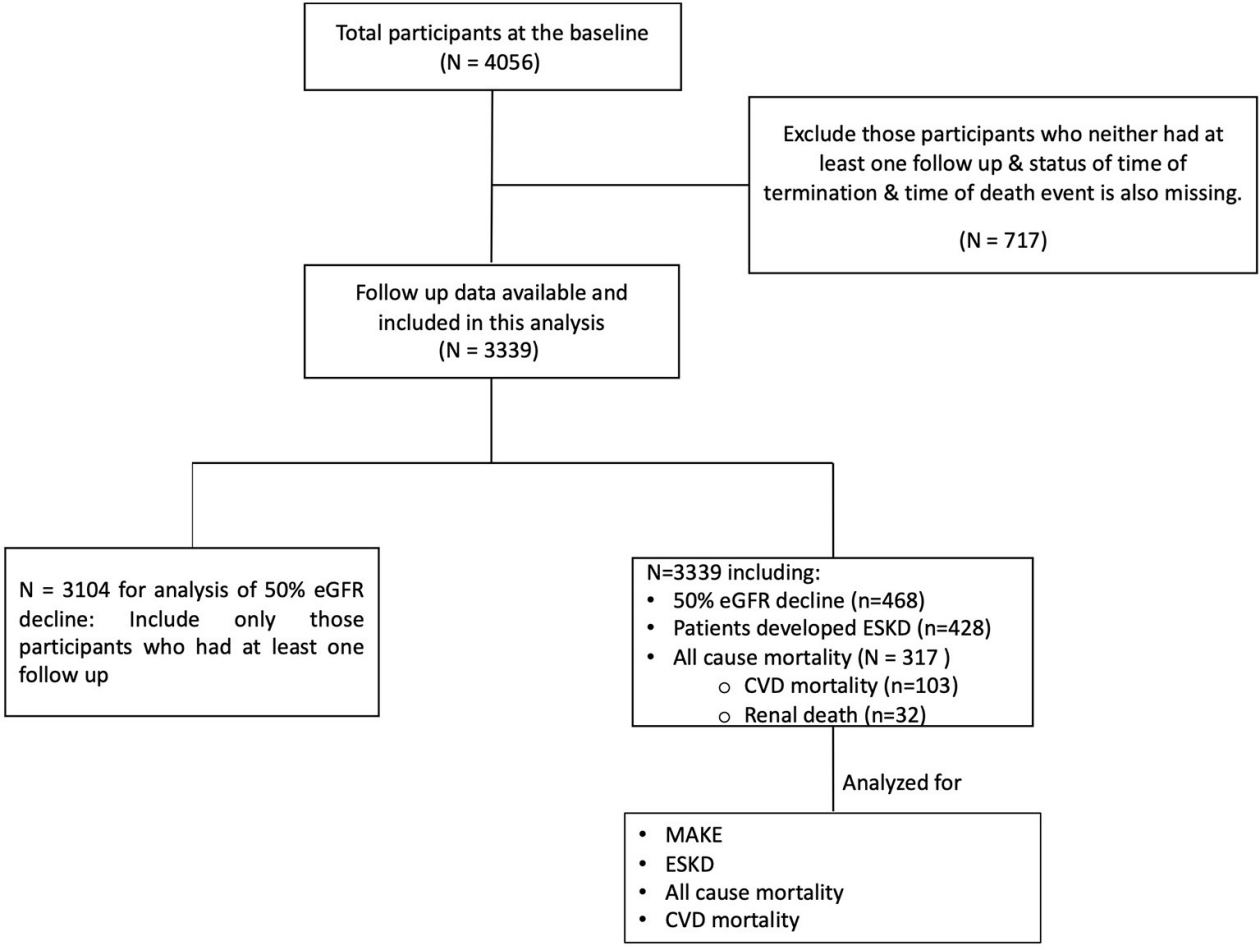
Flow diagram showing study participants.

Flow chart of the data analyzed in this study have been mentioned in Figure 1. During a median follow-up duration of 2.64 (1.40, 3.89) years, mean eGFR decline was 2.47 (2.05, 2.88) ml/min/1.73 m^2^. Out of 3,339 participants with available follow up data, MAKE event was observed in 622 (18.63%) and ESKD was observed in 428 (12.83%) participants. Among 3,104 participants for whom eGFR was available at last follow up, 468 (15.08%) experienced ≥50% eGFR decline. Overall mortality and cardiovascular mortality were reported in 317 (9.49%) and 103 (3.08%) participants out of 3,339 for whom follow-up data were available (Table 3).

**Table 3:** Outcome events in ICKD cohort at follow-up with median duration of 2.64 (1.40, 3.89) years.

**Outcome**	**Females**	**Males**	**Total**
MAKE (50%GFR decline/ESKD/renal deaths) (*N* = 3,339)^*^	225 (20.33)	397 (17.79)	622 (18.63)
50% eGFR decline (*N* = 3,104)^*^	173 (16.80)	295 (14.22)	468 (15.08)
ESKD (eGFR < 15 ml/min or KRT) (*N* = 3,339)^*^	159 (14.38)	269 (12.07)	428 (12.83)
All-cause mortality (*N* = 3,339)^*^	106 (9.58)	211 (9.45)	317 (9.49)
CVD mortality (*N* = 3,339)^*^	25 (2.26)	78 (3.49)	103 (3.08)

MAKE, major adverse kidney events; eGFR, estimated glomerular filtration; ESKD, end stage kidney disease; KRT, kidney replacement therapy; CVD, cardiovascular disease.

* number of subjects with follow-ups.

### Predictors of the use of ACEI/ARBs

Supplementary Table 2 shows the factors associated with the use of ACEI or ARBs at baseline. The odds ratio of the use of these agents decreased with advancing CKD stages. The elderly (age ≥60 years) and males were less likely to get ACEI/ARBs. The unadjusted ratio suggested that urban people had more chances of getting angiotensin-blocking agents. However, the adjusted odds were not significant. The household income did not affect ACEI/ARB use, but educated people were more likely and those with lower BMI were less likely to get ACEI/ARB. ACEI/ARBs use was greater amongst diabetics, and patients with albuminuria. Patients on statins and diuretics were higher likely to get ACEI/ARBs whereas those on BBs and CCBs had lesser likelihood to get ACEI/ARBs. Patients with systolic BP ≥140 had higher chance of using ACEI/ARBs.

In a separate multivariable analysis (Supplementary Table 3), we examined the predictors of the use of ACEI/ARBs by CKD stage. The significant findings included greater use of ACEIs/ARBs amongst diabetics, those with systolic BP ≥140, and in those with albuminuria. Statin and diuretic users had a greater odds of getting ACEI/ARBs in CKD stage 3 whereas those on BBs were less likely to use them. With the progression of CKD stages, patients with albuminuria and those on statins were more likely to receive ACEI/ARBs. Diabetic patients had a higher likelihood of being prescribed ACEI/ARBs in advanced CKD stages (3 and 4).

### Impact of angiotensin pathway blockers on EGFR decline

**Figure 2:**
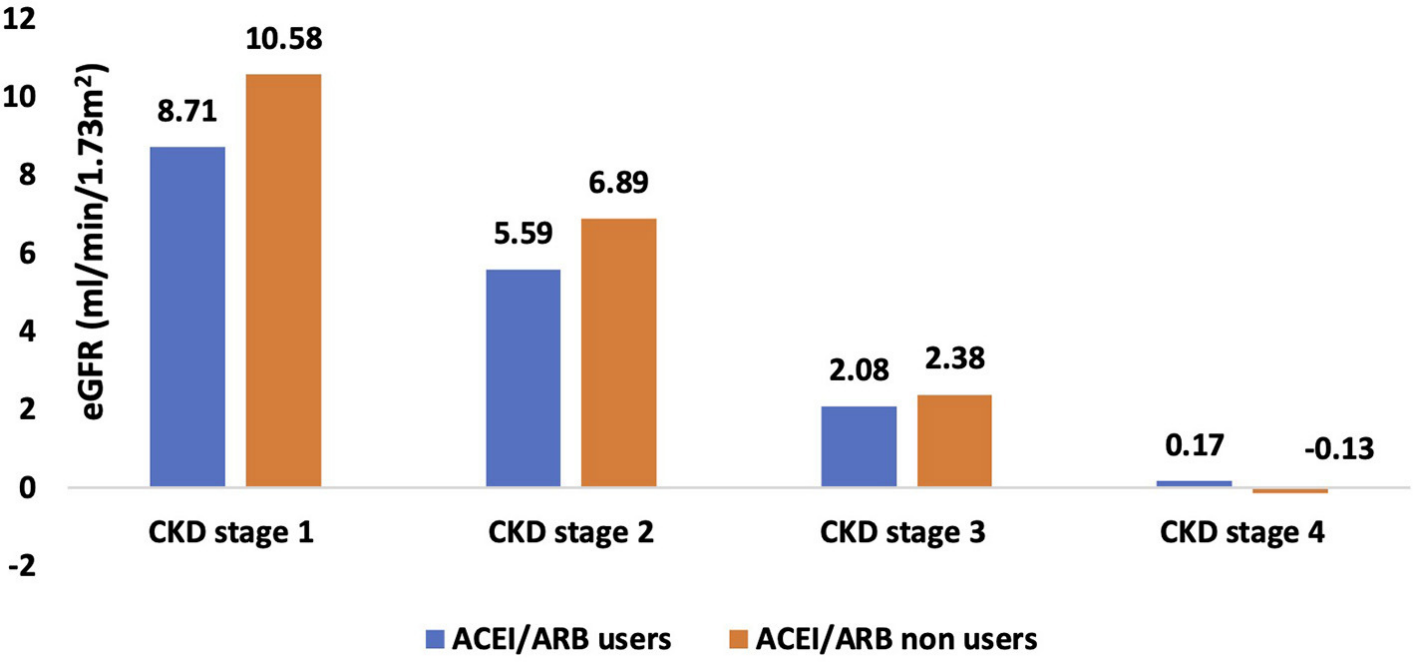
Bar graph representing annual mean eGFR decline in CKD stage 1–4 in ICKD cohort.

Figure 2, Supplementary Table 4 show details of the annual rate of decline in eGFR amongst ACEI/ARB users and nonusers. The overall annual rate of decline in eGFR in the ACEI/ARB users was 2.63 ml/min/1.73 m^2^ as compared to 2.40 ml/min/1.73 m^2^ in the nonusers. The annual rate of decline in eGFR in stage 1 CKD was 10.58 ml/min/1.73 m^2^ in ACEI/ARB nonusers and 8.71 ml/min/1.73 m^2^ in users. The rate of eGFR decline in those with stage 2 CKD was 6.89 and 5.59 ml/min/1.73 m^2^ in ARB nonusers and users respectively. In those with stage 3 disease the nonuser and users showed a rate of decline of 2.38 and 2.08 ml/min/1.73 m^2^ respectively. Finally, the eGFR decline was 0.17 in users and−0.13 ml/min/1.73 m^2^ in nonusers of ARB in stage 4 CKD.

### Risk of adverse kidney events based on anti-hypertensive use

According to unadjusted regression, compared to nonusers, ACEI/ARB users had a significantly lower risk of developing a MAKE (unadjusted SHR= 0.82, 95% CI: 0.70, 0.97, Table 4). After adjustment, the association between ACEIs/ARBs usage and MAKE became non-significant. In addition, no statistically significant association was found between diuretic use and risk of MAKE. Other anti-hypertensives, e.g., CCBs, BBs, alpha-blockers, and central sympatholytic were not significantly associated with MAKE (Supplementary Table 5). When the components of MAKE were analyzed individually, ACEIs/ARBs users had a lower likelihood of experiencing ≥50% decline in eGFR [adjusted SHR = 0.80, 95% CI: 0.64, 0.99], and ESKD [adjusted SHR = 0.72, 95% CI: 0.58, 0.90] (Table 4). The risk of cardiovascular mortality [adjusted SHR = 0.55, 95% CI: 0.34, 0.88] was lower amongst those who were on ACEI/ARB (Table 4). Diuretic use was associated with an increased risk of all-cause mortality [adjusted HR = 1.95, 95% CI: 1.50, 2.53] and cardiovascular mortality (adjusted SHR = 1.73, 95% CI: 1.09, 2.73] (Table 4). Other antihypertensives did not show any statistically significant association with any of the end points (Supplementary Tables 6–9).

**Table 4:** Risk of adverse outcomes associated with the use of ACEI/ARBs and diuretics in the ICKD cohort.

	**Unadjusted sub-hazard ratio (95% CI)**	***P* value**	**Adjusted sub-hazard ratio (95% CI)**	***P* value**
**ACEI/ARBs**				
MAKE	0.82 (0.70, 0.97)	0.02	0.85 (0.71, 1.02)	0.07
50% eGFR decline	0.78 (0.65, 0.93)	0.01	0.80 (0.64, 0.99)	0.04
ESKD	0.73 (0.60, 0.89)	<0.01	0.72 (0.58, 0.90)	<0.01
CVD mortality	0.95 (0.65, 1.39)	0.78	0.55 (0.34, 0.88)	0.01
All-cause mortality^*^	0.96 (0.77, 1.21)	0.76	0.77 (0.59, 1.00)	0.05
**Diuretics**				
MAKE	1.12 (0.95, 1.33)	0.18	1.11 (0.92, 1.34)	0.29
50% eGFR decline	1.08 (0.89, 1.31)	0.44	1.09 (0.87, 1.35)	0.46
ESKD	1.24 (1.01, 1.52)	0.04	1.24 (0.99, 1.56)	0.07
CVD mortality	2.79 (1.88, 4.15)	<0.01	1.73 (1.09, 2.73)	0.02
All-cause mortality^*^	2.22 (1.77, 2.79)	<0.01	1.95 (1.50, 2.53)	<0.01

Adjusted for baseline age, residence, sex, income, any sign of heart failure, eGFR, baseline albuminuria, diabetes mellitus, obesity, systolic BP, aspirin use, statin use, family history of stroke.

MAKE, major adverse kidney events; eGFR, estimated glomerular filtration; ESKD, end stage kidney disease; CVD, cardiovascular disease; ACEI, angiotensin converting enzyme inhibitor; ARB, angiotensin receptor blocker.

* Hazard ratio is presented for All-cause mortality.

For CVD mortality as an outcome of interest, death due to any other cause is considered as competing event. For 50% GFR decline, ESRD and MAKE as outcomes of interest, non-renal death is considered as competing event. Hence, the sub-hazard ratios (SHR) reported for these outcomes.

## Discussion

In this large prospective cohort study, we observed that ACEI/ARB use decreased and the use of CCBs, BBs, and diuretics increased with advancing CKD stages. Many patients, despite being hypertensive, were not receiving ACEI/ARB in the early stages of CKD despite clear recommendations by all major clinical practice guidelines (14–16). The study confirmed the reduced risk of eGFR decline, development of ESKD and cardiovascular mortality in ACEI/ARB users as compared to nonusers. The use of diuretics was associated with higher risk of mortality. These findings were consistent, even after adjusting for multiple variables like age, residence, sex, income, eGFR, albuminuria, diabetes mellitus, obesity, blood pressure, aspirin use, statin use, presence of heart failure, and history of stroke, which affect the survival of the patients. This is the first study from any developing country to show these findings. Further, our study establishes the fact that amongst diabetics, those with systolic BP ≥140, and patients with albuminuria are likely to receive ACEI/ARBs in CKD stage 3.

Similar to our study, a sub-study of CRIC cohort showed that the use of BBs, CCBs, and diuretics steadily increased, whereas the use of ACEI/ARB decreased with advancing stages of CKD (11). In that study, during a median follow-up of 7.5 years, RAASi use plateaued during CKD stage 3 (75%) and declined to 37% by stage 5, while BB, CCB, and diuretic use increased steadily with advancing CKD. These agents were prescribed to 46.8% of stage 3 and 30.5% of stage 4 CKD patients in our cohort, despite the known reno-protective effect of RAAS blockers (17–19).

The cardiovascular protection conferred by ACEI/ARBs across all stages of CKD has been confirmed in several systematic reviews and observational studies (summarized in Table 5). However, there are uncertainties around the use of ACEI/ARB in the more advanced stages of CKD. When and whether to stop ACEI/ARB on longitudinal follow-up of CKD patients is not clear (20–22). Although the use of ACEI/ARB was associated with benefits on multiple clinically important endpoints overall, this was not seen in the group with stage 4 CKD, in which there was a numerically greater decline in eGFR amongst the RAASi users. However, the number of patients in stage 4 CKD was relatively small. Similar findings have been reported by others (22–24). It has been suggested that continuing ACEI/ARB use in patients with advanced CKD might accelerate the need for kidney replacement therapy (KRT) (25). A recently published nationwide observational study of 10,254 prevalent RAS inhibitor users with new-onset eGFR <30 ml/min per 1.73 m^2^ from Sweden (23) showed that stopping RAS inhibition was associated with increased risks of mortality and major adverse cardiovascular events, but also with a lower absolute risk of initiating KRT. An observational study from the United States suggested that stopping ACEI/ARBs in patients with advanced CKD was associated with an increased risk of major cardiovascular events and death, but not with the risk of KRT (24). However, Qiao et al. observed that continuing ACEI/ARBs was not associated with increased risk of RRT and they emphasized that the KRT-related harms may not be excessive, as stopping ACEI/ARBs may also harm patients by increasing cardiovascular risk and mortality (26, 27). A recent retrospective study from the USA also did not find differences in risk for progression to ESKD or mortality based on patterns of RAS inhibitor use during advanced stages of CKD (28). Finally, the recent STOP-ACEi study that randomized 411 patients with eGFR <30 ml/min/1.73 m^2^ did not find any difference in the long-term rate of decrease in the eGFR following the discontinuation of RAS inhibitors (29). The higher risk of cardiovascular death with diuretics may be associated with volume overload and congestive failure in these patients (30, 31). The exact dose of these agents that provides optimal benefit is not known, but clinical practice guidelines recommend using the maximally tolerated dose (32).

**Table 5:** Major systematic review and randomized study which compared ACEI/ARB against placebo or active control population with other anti-hypertensives.

	**Study/subjects**	**Drugs**	**Compared to placebo**	**Compared to active groups**	**Remarks**
Zhang et al. (7)	RCTs in non-dialysis CKD3–5 patients Forty-four randomized clinical trials with 42,319 patients were included in our network meta-analysis.	ACEI monotherapy With placebo and comparator other anti-hypertensives	decreased the odds of kidney events, cardiovascular events, cardiovascular death, and all-cause death	No significant differences between ACEIs and other antihypertensive drugs, including CCBs, BB, and diuretics on other outcomes except for kidney events	Also included stage V CKD
Zhao et al. (33)	The meta-analysis includes available evidences to compare the effect of CCBs and ACEIs or ARBs on renal outcomes and mortality. Eight clinical trials, 25,647 participants	CCB plus ACEI/ARB	No placebo	ESKD is higher with CCB. BP decreases similarly. CCBs did not increase all-cause mortality incidence in patients with CKD though they displayed weaker reno protective, compared to ACEIs or ARBs therapy.	
Lin et al. (34)	Systematic review and meta-analysis comparing CCBs and the two RAAS blockades for hypertensive patients with CKD stage 3 to 5D 21RCTs randomized 9,492 patients	CCB plus ACEI/ARB	No placebo	CCBs have similar effects on long-term BP, mortality, heart failure, stroke or cerebrovascular events, and renal function to RAAS blockades in patients CKD stage 3 to 5D and hypertension.	Stage 3 to 5 D
Xie et al. (37)	A Bayesian Network Meta-analysis of RCTs (*n* = 119) Participants (*n* = 64,768)	ACEI/ARB vs. placebo and active control	ACEI/ARB reduced OR for kidney failure by 39% and 30% compared to placebo. whereas other active controls did not show evidence of a significant effect on kidney failure. ACEI/ARB reduced OR for major cardiovascular events vs. placebo, but not for cardiovascular death.	Reduced OR for kidney failure by 35% and 25% compared with other active controls. ARBs, ACE inhibitors were consistently associated with higher probabilities of reducing kidney failure, cardiovascular death, or all-cause death.	ACE inhibitors but not ARBs significantly reduced the odds of all-cause death vs. active controls. Included all CKD patients including dialysis
Wu et al. (38)	A systematic review and Bayesian network meta-analysis 63 trials with 36,917 participants.	RCTs of ACEI, ARBs, α blockers, BBs, CCB, diuretics, and their combinations Placebo as well as active control	Compared with placebo, only ACEI significantly reduced the doubling of serum creatinine levels and only BB showed high mortality.	The protective effect of an ACEI plus CCB compared with a placebo was not statistically significant. ACEI plus CCB was the best treatment for reducing mortality, followed by ACEI plus diuretic.	
Ku et al. (11)	3,939 participants of the CRIC (Chronic Renal Insufficiency Cohort) study	Observational study	Outcomes with users of ACEI/ARB compared with beta-blocker, and calcium channel blocker	ACEI/ARB was associated with lower risk of death across all stages of CKD. Beta blockers were associated with higher risk of death. CCB was not associated with risk of death.	All CKD stages

CCB, calcium channel blocker; ACEI, angiotensin-converting enzyme inhibitor; ARB, angiotensin receptor blocker; CKD, chronic kidney disease; BB, beta blocker; RCT, randomized clinical trial; ESKD, end stage kidney disease.

The effect of other antihypertensives was neutral with regard to the end points studied. Similar to our study, CCBs did not increase all-cause mortality incidence in patients with CKD and showed a weak reno-protective effect, compared to ACEI/ARBs (33). A systematic review and meta-analysis showed that CCBs had similar effects on long-term blood pressure control, mortality, heart failure, stroke or cerebrovascular events and kidney function to RAAS blockades in patients CKD stage 3 to 5D (34). In our study, in interaction with the use of different classes of anti-hypertensives, ACEI/ARB users had a significantly lower risk of cardiovascular mortality, confirming that the choice of anti-hypersensitive to modulate these effects of paramount importance in the Indian population as well (35).

The major strength of our study is prospectively collected data of ICKD cohort, and the comparison of all anti-hypertensive used in the management of hypertension in CKD patients. Besides kidney outcomes, we also analyzed all cause- mortality and cardiovascular mortality and adjusted for all variable affecting the outcome. This is the only data available from this part of the developing world. Our study has some limitations, however. These include relatively short duration of follow-up and a small number of patients with stage 4 CKD. About 17% of patients were lost to follow-up, which could have caused bias. We were also unable to determine the overall exposure to ACEI/ARBs in relation to the treatment duration or dose effect. We also cannot comment on the reasons for the low ACEI/ARB use. Low-cost generic preparations of these agents are widely available in India. Although we have adjusted the analysis for a range of factors, the effect of unmeasured confounders cannot be totally ruled out. However, the impact on the outcome with the use of ACEI/ARB users was huge, even on short follow-up, and emphasizes the need to encourage the use of ACEI/ARB in early CKD. During the COVID pandemic, there has been a debate on the ability of ARBs to protect against development of severe disease. Our data, however, was collected mostly before the pandemic (36).

In conclusion, the use of ACEI/ARBs decreased with advancing CKD stages from stage 1 to 4. ACEI/ARB users exhibited a slower rate of decline in eGFR, especially in CKD stages 1–3, with a neutral effect in stage 4. However, they had a significantly lower risk of cardiovascular mortality across all stages. Diuretic use was associated with higher mortality in our cohort. Use of agents that block angiotensin pathway is reno-protective and cardioprotective in patients with CKD.

## Data availability statement

The original contributions presented in the study are included in the article/Supplementary material, further inquiries can be directed to the corresponding author.

## Ethics statement

The studies involving human participants were reviewed and approved by Institute Ethics Committee, Postgraduate Institute of Medical Education and Research, Chandigarh, India. The patients/participants provided their written informed consent to participate in this study.

## Author contributions

NP, AY, and VJ conceptualized the study. AY and VK performed data curation. MK and AG analyzed the data. AJ, DS, GM, MS, NG, SVi, SVa, SS, SG, SP, and VK verified clinical data and investigations. NP and AY drafted the original manuscript. VJ and SB-A supervised the study. VJ reviewed and edited the manuscript. All authors reviewed and approved the final version.
