# Supplementary material for: Renin-angiotensin blocker use is associated with improved cardiovascular mortality in Indian patients with mild-moderate chronic kidney disease—findings from the ICKD study
Source: Front Med (Lausanne). 2022 Dec 20;9:1060148. doi: 10.3389/fmed.2022.1060148 (PMC9807808; doi:10.3389/fmed.2022.1060148)
Supplement: Supplementary file 1 [file Data_Sheet_1.PDF]

Supplementary table 1: Baseline characteristics of CKD participants as per CKD stages in ICKD cohort

**N = 4056**	**CKD stage 1**<br>**N = 104 (2.56)**	**CKD stage 2**<br>**N = 318 (7.84)**	**CKD stage 3**<br>**N = 3190 (78.65)**	**CKD stage 4**<br>**N = 444 (10.95)**
**Age (years)**<br>Mean (SD)	36.66 (11.70)	44.50 (11.96)	50.93 (11.47)	53.15 (10.71)
**Sex**<br>Female<br>Male	 39 (37.50)<br>65 (62.50)	 60 (18.87)<br>258 (81.13)	 948 (29.72)<br>2242 (70.28)	 284 (63.96)<br>160 (36.04)
**Residence**<br>Rural<br>Urban	 56 (53.85)<br>48 (46.15)	 178 (56.69)<br>136 (43.31)	 2,102 (67.05)<br>1033 (32.95)	 290 (68.56)<br>133 (31.44)
**Annual household income**<br>Quartile-1<br>Quartile-2<br>Quartile-3<br>Quartile 4	25 (24.04)<br>14 (13.46)<br>29 (27.88)<br>36 (34.62)	73 (23.03)<br>70 (22.08)<br>85 (26.81)<br>89 (28.08)	881 (27.90)<br>731 (23.15)<br>794 (25.14)<br>752 (23.81)	143 (32.80)<br>100 (22.94)<br>100 (22.94)<br>93 (21.33)
**Education level**<br>Uneducated<br>Below high school<br>Completed school<br>College and above	13 (12.50)<br>29 (27.88)<br>22 (21.15)<br>40 (38.46)	55 (17.35)<br>96 (30.28)<br>50 (15.77)<br>116 (36.59)	841 (26.49)<br>1112 (35.02)<br>412 (12.98)<br>810 (25.51)	179 (40.50)<br>137 (31.00)<br>54 (12.22)<br>72 (16.29)
**ACEI or ARB use**	72 (72.00)	182 (58.90)	1465 (46.79)	130 (30.52)
**BMI**<br>>=25<br>18-24.9<br><18	 44 (42.72)<br>50 (48.54)<br>9 (8.74)	 132 (41.64)<br>166 (52.37)<br>19 (5.99)	 1372 (44.22)<br>1566 (50.47)<br>165 (5.32)	 193 (44.88)<br>206 (47.91)<br>31 (7.21)
**Diabetes**	17 (17)	93 (30.29)	1215 (38.86)	160 (37.56)
**Hypertension**	92 (92)	275 (88.42)	2742 (86.83)	378 (86.30)
**Systolic blood pressure >=140**	27 (26.21)	122 (38.98)	1217 (39.17)	189 (43.75)
**Diastolic blood pressure >=90**	27 (26.21)	113 (36.45)	990 (32.11)	167 (39.11)
**Cardiovascular diseases (CVD)**	10 (9.62)	48 (15.14)	706 (22.29)	112 (25.75)
**Albuminuria**	38 (38.00)	67 (22.41)	745 (24.92)	117 (29.62)
**Statin use**	27 (27.00)	110 (35.60)	1312 (41.90)	153 (35.92)

**B-Blocker use**	13 (13.00)	64 (20.71)	881 (28.14)	118 (27.70)
**Ca-channel blocker use**	18 (18.00)	119 (38.51)	1348 (43.05)	203 (47.65)
**Diuretic use**	14 (14.00)	70 (22.65)	905 (28.90)	148 (34.74)
**Alpha blocker**	7 (7.00)	26 (8.41)	349 (11.15)	40 (9.39)
**Central sympatholytic**	2 (2.00)	6 (1.94)	93 (2.97)	17 (3.99)

Supplementary table 2: Unadjusted and adjusted logistic regression results for ACEI/ARB use at baseline.

	**Unadjusted OR (95%CI)**	***P-value***	**Adjusted OR (95%CI)<br>N = 3442**	***P-value***
**CKD stages**<br>**1®**<br>**2**<br>**3**<br>**4**	 0.56 (0.34, 0.91)<br>0.34 (0.22, 0.53)<br>0.17 (0.11, 0.28)	 0.02<br><0.01<br><0.01	 0.58 (0.34, 0.98)<br>0.35 (0.22, 0.57)<br>0.17 (0.10, 0.28)	 0.04<br><0.01<br><0.01
**Age (years)**<br>< 60 years®<br>>= 60 years	 0.68 (0.59, 0.79)	 <0.01	0.62 (0.53, 0.74)	<0.01
**Sex**<br>Female®<br>Male	 0.97 (0.85, 1.10)	 0.62	0.82 (0.69, 0.97)	0.02
**Residence**<br>Rural®<br>Urban	 1.27 (1.11, 1.45)	 <0.01	1.03 (0.87, 1.20)	0.76
**Annual household income (USD)**<br>Quartile-1®<br>Quartile-2<br>Quartile-3<br>Quartile 4	1.00 (0.84, 1.20)<br>1.01 (0.85, 1.20)<br>1.12 (0.94, 1.33)	0.99<br>0.93<br>0.21	1.0 (0.82, 1.23)<br>0.91 (0.74, 1.12)<br>0.82 (0.65, 1.03)	0.97<br>0.39<br>0.08
**Education level**<br>Uneducated®<br>Below high school<br>Completed school<br>College and above	 <br>1.35 (1.15, 1.59)<br>1.65 (1.34, 2.04)<br>1.47 (1.24, 1.75)	 <br><0.01<br><0.01<br><0.01	 <br>1.27 (1.05, 1.54)<br>1.85 (1.44, 2.40)<br>1.78 (1.39, 2.27)	 <br>0.02<br><0.01<br><0.01
**BMI**<br>>=25®<br>18-24.9<br><18	 0.69 (0.61, 0.79)<br>0.43 (0.32, 0.58)	<0.01<br><0.01	0.73 (0.63, 0.85)<br>0.53 (0.38, 0.76)	<0.01<br><0.01
**Diabetes**<br>No®<br>Yes	 <br>1.80 (1.58, 2.05)	<0.01	1.31 (1.11, 1.54)	<0.01
**Systolic blood pressure**<br><140®<br>>=140	 1.30 (1.14, 1.48)	<0.01	1.27 (1.06, 1.52)	0.01
**Diastolic blood pressure**<br><90®<br>>=90	1.09 (0.95, 1.25)	0.21	1.05 (0.87, 1.26)	0.63

**Cardiovascular diseases (CVD)**<br>No®<br>Yes	1.15 (0.99, 1.34)	0.07	0.99 (0.82, 1.19)	0.89
**Albuminuria**	1.85 (1.59, 2.15)	<0.01	1.49 (1.26, 1.76)	<0.01
**Statin use**<br>No®<br>Yes	2.91 (2.55, 3.32)	<0.01	2.67 (2.28, 3.12)	<0.01
**B-Blocker use**<br>No®<br>Yes	0.74 (0.64, 0.85)	<0.01	0.67 (0.57, 0.80)	<0.01
**Ca-channel blocker use**<br>No®<br>Yes	0.86 (0.76, 0.98)	0.02	0.75 (0.64, 0.87)	<0.01
**Diuretic use**<br>No®<br>Yes	1.29 (1.13, 1.48)	<0.01	1.20 (1.02, 1.42)	0.03

Supplementary table 3: Predictors of ACEI/ARB use as the outcome of interest by each CKD stage

	**CKD stage 1**<br>**N = 160**		**CKD stage 2**<br>**N = 464**		**CKD stage 3**<br>**N = 2884**		**CKD stage 4**<br>**N = 1002**	
	**OR (95% CI)**							
		P value		*P value*		*P value*		*P value*
**Age (years)**<br>< 60 years®<br>>= 60 years	0.08 (0.02, 0.45)	<0.01	0.71 (0.41, 1.22)	0.22	0.62 (0.51, 0.74)	<0.01	0.53 (0.39, 0.74)	<0.01
**Sex**<br>Female®<br>Male	2.50 (1.03, 6.05)	0.04	0.78 (0.47, 1.30)	0.34	0.80 (0.67, 0.96)	0.02	1.02 (0.76, 1.36)	0.90
**Residence**<br>Rural®<br>Urban	2.08 (0.91, 4.76)	0.09	0.78 (0.51, 1.19)	0.25	1.05 (0.88, 1.25)	0.57	0.80 (0.59, 1.09)	0.16
**Annual household income (USD)**<br>Quartile-1®<br>Quartile-2<br>Quartile-3<br>Quartile 4	0.16 (0.03, 0.83)<br>0.15 (0.03, 0.62)<br>0.12 (0.03, 0.53)	0.03<br>0.01<br>0.01	1.46 (0.79, 2.69)<br>1.00 (0.55, 1.82)<br>0.81 (0.43, 1.55)	0.23<br>1.00<br>0.53	1.06 (0.85, 1.33)<br>1.01 (0.80, 1.27)<br>0.88 (0.69, 1.14)	0.61<br>0.95<br>0.34	0.98 (0.66, 1.45)<br>0.70 (0.47, 1.06)<br>0.80 (0.59, 1.09)	0.92<br>0.09<br>0.35
**Education level**<br>Uneducated®<br>Below high school<br>Completed school<br>College and above	1.13 (0.25, 5.02)<br>4.86 (1.03, 22.88)<br>3.34 (0.76, 14.76)	0.88<br>0.05<br>0.11	1.56 (0.84, 2.88)<br>2.44 (1.17, 5.07)<br>2.62 (1.28, 5.37)	0.16<br>0.02<br>0.01	1.26 (1.02, 1.56)<br>1.73 (1.31, 2.29)<br>1.54 (1.19, 2.01)	0.03<br><0.01<br><0.01	1.37 (0.95, 1.97)<br>1.75 (1.06, 2.90)<br>1.30 (0.81, 2.10)	0.09<br>0.03<br>0.28
**BMI**<br>>=25®<br>18-24.9<br><18	1.18 (0.49, 2.86)<br>0.70 (0.15, 3.35)	0.71<br>0.66	0.69 (0.45, 1.05)<br>0.40 (0.16, 0.99)	0.08<br>0.05	0.74 (0.63, 0.87)<br>0.53 (0.35, 0.79)	<0.01<br><0.01	0.64 (0.48, 0.86)<br>0.53 (0.28, 1.02)	<0.01<br>0.06

**Diabetes**<br>No®<br>Yes	1.57 (0.51, 4.80)	0.43	1.08 (0.67, 1.75)	0.74	1.35 (1.14, 1.61)	<0.01	1.44 (1.06, 1.95)	0.02
**Systolic blood pressure**<br><140®<br>>=140	2.63 (0.74, 9.34)	0.13	1.31 (0.78, 2.21)	0.31	1.28 (1.06, 1.55)	0.01	1.30 (0.92, 1.83)	0.13
**Diastolic blood pressure**<br><90®<br>>=90	1.55 (0.47, 5.16)	0.47	0.78 (0.47, 1.30)	0.35	1.06 (0.87, 1.30)	0.55	0.79 (0.56, 1.13)	0.20
**Cardiovascular diseases (CVD)**<br>No®<br>Yes	0.89 (0.22, 3.53)	0.87	0.86 (0.50, 1.47)	0.58	1.00 (0.82, 1.22)	0.97	0.97 (0.69, 1.36)	0.85
**Albuminuria**	0.84 (0.35, 2.02)	0.70	2.07 (1.22, 3.51)	0.01	1.59 (1.32, 1.91)	<0.01	1.38 (1.01, 1.88)	0.04
**Statin use**<br>No®<br>Yes	1.38 (0.51, 3.73)	0.52	2.47 (1.59, 3.83)	<0.01	2.60 (2.19, 3.07)	<0.01	3.11 (2.32, 4.17)	<0.01
**B-Blocker use**<br>No®<br>Yes	0.67 (0.23, 2.01)	0.48	0.81 (0.50, 1.30)	0.38	0.68 (0.57, 0.82)	<0.01	0.78 (0.56, 1.08)	0.14
**Ca-channel blocker use**<br>No®<br>Yes	0.21 (0.07, 0.66)	0.01	1.17 (0.76, 1.78)	0.48	0.77 (0.65, 0.90)	<0.01	0.82 (0.61, 1.11)	0.20
**Diuretic use**<br>No®<br>Yes	1.97 (0.51, 7.51)	0.32	0.62 (0.38, 1.00)	0.05	1.33 (1.11, 1.60)	<0.01	1.07 (0.79, 1.44)	0.68

Supplementary Table 4: eGFR at baseline and follow-up in various subgroups.

	Baseline	12M	24M	36M	48M	Last FU
Stage 1						
RAASB users N(%)	72 (72)	19 (73.08)	9 (69.23)	3 (42.86)	4 (80)	37 (68.52)
Nonusers N(%)	28 (28)	7 (26.92)	4 (30.77)	4 (57.14)	1 (20)	17 (31.48)
eGFR in RAASB users	107.33 (12.29)	92.65 (20.39)	83.04 (27.95)	79.36 (29.53)	89.00 (24.78)	85.83 (26.30)
eGFR in RAASB non-users	108.94 (14.43)	94.20 (21.27)	82.79 (27.78)	87.91 (31.91)	88.48 (38.46)	83.53 (27.02)
Overall eGFR Mean (sd)	107.64 (13.20)	93.01 (20.43)	82.97 (27.60)	82.29 (30.18)	88.88 (27.72)	85.21 (26.36)
eGFR in albuminuric	108.65 (12.26)	93.75 (21.31)	85.29 (25.64)	83.67 (30.89)	88.37 (30.45)	87.69 (26.34)
eGFR in Non-albumin uric	105.27 (11.96)	91.93 (19.47)	76.42 (31.43)	77.63 (29.10)	89.97 (22.67)	80.74 (26.38)
eGFR in Diabetics	101.98 (9.67)	79.37 (17.15)	77.28 (30.37)	73.63 (34.15)	82.44 (25.96)	79.94 (28.06)
eGFR Non-diabetics	108.97 (13.16)	94.77 (20.28)	84.14 (27.28)	84.09 (29.63)	90.49 (28.56)	86.31 (26.06)
Stage 2						
RAASB users N(%)	182 (58.90)	36 (53.73)	23 (41.82)	18 (41.86)	11 (50)	81 (48.80)
Nonusers N(%)	127 (41.10)	31 (46.27)	32 (58.18)	25 (58.14)	11 (50)	85 (51.20)
eGFR in RAASB users	70.13 (8.06)	63.96 (19.07)	62.89 (18.52)	62.64 (15.41)	63.11 (23.06)	59.25 (22.07)
eGFR in RAASB non-users	69.56 (8.30)	58.60 (23.45)	52.86 (22.15)	51.39 (21.79)	54.11 (23.59)	53.16 (24.10)

Overall eGFR Mean (sd)	69.83 (8.13)	61.73 (20.92)	58.84 (20.57)	58.08 (18.91)	60.57 (23.27)	56.49 (23.25)
eGFR in albuminuric	70.14 (8.54)	61.49 (20.64)	56.94 (18.14)	57.43 (17.24)	62.12 (23.09)	56.35 (23.84)
eGFR in Non-albumin uric	69.63 (8.06)	61.64 (21.39)	60.69 (23.56)	58.79 (21.14)	59.10 (23.93)	57.46 (22.92)
eGFR in Diabetics	67.71 (7.26)	55.06 (17.11)	54.73 (22.53)	57.23 (20.99)	50.39 (27.83)	52.82 (21.73)
eGFR Non-diabetics	70.84 (8.38)	64.57 (21.60)	61.02 (19.18)	58.35 (18.25)	63.63 (21.30)	58.69 (23.30)
Stage 3						
RAASB users N(%)	1465 (46.79)	373 (47.10)	295 (46.60)	208 (46.02)	126 (44.21)	854 (43.35)
Nonusers N(%)	1666 (53.21)	419 (52.90)	338 (53.40)	244 (53.98)	159 (55.79)	1116 (56.65)
eGFR in RAASB users	43.01 (8.28)	40.67 (14.89)	41.84 (17.98)	40.74 (18.78)	44.19 (23.28)	39.74 (20.57)
eGFR in RAASB non-users	41.20 (7.94)	38.69 (15.96)	38.94 (18.36)	41.00 (22.37)	41.28 (24.85)	37.67 (21.43)
Overall eGFR Mean (sd)	42.02 (8.16)	39.57 (15.68)	40.20 (18.23)	40.84 (20.74)	42.55 (24.20)	38.58 (21.06)
eGFR in albuminuric	42.01 (8.17)	38.89 (14.76)	39.39 (17.79)	39.12 (19.26)	40.90 (23.22)	37.93 (21.06)
eGFR in Non-albumin uric	42.24 (8.23)	40.54 (16.37)	41.25 (18.81)	42.98 (22.28)	44.37 (25.17)	39.32 (21.05)
eGFR in Diabetics	41.98 (8.28)	38.25 (15.96)	38.59 (18.11)	40.71 (19.49)	42.10 (24.11)	37.70 (20.74)
eGFR Non-diabetics	42.09 (8.08)	40.31 (15.25)	40.91 (18.28)	40.81 (21.32)	42.70 (24.38)	39.16 (21.27)

Stage 4						
RAASB users N(%)	130 (30.52)	31 (27.43)	18 (23.08)	14 (28)	9 (30)	65 (23.72)
Nonusers N(%)	296 (69.48)	82 (72.57)	60 (76.92)	36 (72)	21 (70)	209 (76.28)
eGFR in RAASB users	26.78 (2.95)	25.99 (8.34)	35.18 (22.21)	28.16 (10.54)	34.48 (20.75)	27.97 (20.40)
eGFR in RAASB non-users	25.94 (3.82)	27.70 (12.67)	29.70 (21.54)	27.56 (23.75)	23.76 (13.12)	26.77 (18.54)
Overall eGFR<br>Mean (sd)	26.09 (3.67)	28.95 (16.61)	30.68 (21.63)	27.70 (21.30)	27.40 (16.72)	27.79 (20.53)
eGFR in albuminuric	26.48 (3.33)	25.30 (9.01)	28.08 (16.56)	23.85 (14.61)	27.72 (14.68)	25.93 (16.24)
eGFR in Non-albuminuric	25.91 (3.89)	31.97 (20.98)	33.30 (25.47)	33.94 (27.47)	29.43 (19.34)	30.17 (23.90)
eGFR in Diabetics	26.57 (3.10)	25.34 (8.39)	34.82 (22.87)	26.69 (20.57)	29.04 (15.45)	26.79 (18.38)
eGFR Non-diabetics	25.95 (3.86)	28.22 (12.98)	29.36 (21.29)	28.21 (22.02)	26.75 (17.35)	27.29 (19.49)

Table 5: Risk of MAKE associated with the prescription usage and subject characteristics in the ICKD cohort.

	Unadjusted sub-hazard ratio (95% CI)	P value	Adjusted sub-hazard ratio (95% CI)	P value
**ACEI/ARBs**				
No®				
Yes	0.82 (0.70, 0.97)	0.02	0.85 (0.71, 1.02)	0.07
**Diuretics**				
No®				
Yes	1.12 (0.95, 1.33)	0.18	1.11 (0.92, 1.34)	0.29
**Calcium Channel Blockers**				
No®	1.21 (1.03, 1.42)	0.02	1.15 (0.95, 1.39)	
Yes				0.16
**Beta blockers**				
No®	0.97 (0.82, 1.15)	0.74	0.94 (0.76, 1.17)	
Yes				0.59
**Alpha blockers**				
No®				
Yes	1.01 (0.80, 1.26)	0.96	1.23 (0.96, 1.57)	0.10
**Central sympatholytic**				
No®				
Yes	0.10 (0.66, 1.51)	0.99	0.96 (0.58, 1.58)	0.87
**Aspirin**				
No®				
Yes	0.94 (0.77, 1.14)	0.54	0.89 (0.70, 1.15)	0.38
**Statin**				
No®				
Yes	0.76 (0.65, 0.90)	<0.01	0.70 (0.57, 0.85)	0.00
**Age**				
<60®				
60&above	0.89 (0.73, 1.08)	0.24	0.88 (0.71, 1.10)	0.27
**Sex**				
Female®				
Male	0.90 (0.77, 1.06)	0.19	0.97 (0.79, 1.18)	0.76
**Residence**				
Rural®				
Urban	0.80 (0.68, 0.95)	0.01	0.84 (0.69, 1.02)	0.08
**Income quartile (USD annual)**				
Q1: 0-1008®				
Q2: 1176-1680	1.02 (0.80, 1.30)	0.87	1.17 (0.88, 1.55)	0.28
Q3: 1764-4200	0.92 (0.74, 1.16)	0.49	1.09 (0.83, 1.42)	0.55
Q4: 4368-84000	0.89 (0.71, 1.10)	0.29	1.05 (0.80, 1.38)	0.70
**Heart fail**				
No®				
Yes	1.92 (1.02, 3.61)	0.04	1.22 (0.48, 3.13)	0.68
**eGFR**			1.35 (1.03, 1.77)	0.03

>=30®				
<30	1.52 (1.23, 1.89)	<0.01		
**Albuminuria**	1.83 (1.54, 2.16)	<0.01	1.80 (1.48, 2.20)	0.00
**Diabetes**				
No®				
Yes	1.36 (1.15, 1.59)	<0.01	1.39 (1.14, 1.69)	0.00
**Obesity**				
No®				
Yes	0.79 (0.62, 1.01)	0.06	0.77 (0.58, 1.03)	0.08
**Stroke**				
No®				
Yes	0.88 (0.70, 1.10)	0.26	0.87 (0.68, 1.10)	0.25
**Systolic BP**				
<140®				
>=140	1.28 (1.09, 1.49)	<0.01	1.08 (0.90, 1.30)	0.43

MAKE: major adverse kidney events, eGFR: estimated glomerular filtration, ESKD: end stage kidney disease, CVD: cardiovascular disease.

*Hazard ratio is presented for All-cause mortality.

For CVD mortality as an outcome of interest, death due to any other cause is considered as competing event. For 50% GFR decline, ESRD and MAKE as outcomes of interest, non-renal death is considered as competing event. Hence, the sub-hazard ratios (SHR) reported for these outcomes.

Table 6: Risk of 50% eGFR decline associated with the prescription usage and subject characteristics in the ICKD cohort.

	Unadjusted sub-hazard ratio (95% CI)	P value	Adjusted sub-hazard ratio (95% CI)	P value
**ACEI/ARBs**				
No®				
Yes	0.78 (0.65, 0.93)	0.01	0.80 (0.64, 0.99)	0.04
**Diuretics**				
No®				
Yes	1.08 (0.89, 1.31)	0.44	1.09 (0.87, 1.35)	0.46
**Calcium Channel Blockers**				
No®				
Yes	1.17 (0.98, 1.40)	0.09	1.16 (0.93, 1.44)	0.18
**Beta blockers**				
No®				
Yes	0.10 (0.82, 1.21)	0.97	0.95 (0.75, 1.21)	0.68
**Alpha blockers**				
No®				
Yes	1.04 (0.81, 1.34)	0.77	1.30 (0.99, 1.72)	0.06
**Central sympatholytic**				
No®				
Yes	0.97 (0.61, 1.55)	0.90	1.01 (0.59, 1.74)	0.97
**Aspirin**				
No®				
Yes	0.89 (0.71, 1.11)	0.30	0.82 (0.62, 1.09)	0.17
**Statin**				
No®				
Yes	0.78 (0.65, 0.94)	0.01	0.77 (0.62, 0.97)	0.02
**Age**				
<60®				
60&above	0.96 (0.77, 1.19)	0.68	0.98 (0.77, 1.25)	0.88
**Sex**				
Female®				
Male	0.88 (0.73, 1.05)	0.16	0.82 (0.66, 1.03)	0.09
**Residence**				
Rural®				
Urban	0.84 (0.70, 1.02)	0.07	0.84 (0.67, 1.06)	0.14
**Income quartile (USD annual)**				
Q1: 0-1008®				0.08
Q2: 1176-1680	1.17 (0.88, 1.56)	0.27	1.34 (0.96, 1.86)	0.24
Q3: 1764-4200	1.04 (0.80, 1.36)	0.77	1.20 (0.88, 1.64)	0.14
Q4: 4368-84000	1.07 (0.83, 1.39)	0.59	1.27 (0.93, 1.74)	
**Heart fail**				
No®				
Yes	2.23 (1.10, 4.52)	0.03	1.52 (0.53, 4.31)	0.44
**eGFR**				
>=30®	1.06 (0.81, 1.39)	0.68	0.93 (0.66, 1.31)	0.68

<30				
**Albuminuria**	1.74 (1.43, 2.12)	<0.01	1.69 (1.35, 2.11)	0.00
**Diabetes**				
No®				
Yes	1.26 (1.05, 1.52)	0.01	1.36 (1.09, 1.70)	0.01
**Obesity**				
No®				
Yes	0.78 (0.59, 1.04)	0.09	0.80 (0.58, 1.09)	0.16
**Stroke**				
No®				
Yes	1.04 (0.81, 1.33)	0.77	0.97 (0.75, 1.27)	0.84
**Systolic BP**				
<140®				
>=140	1.26 (1.05, 1.51)	0.01	1.12 (0.91, 1.37)	0.31

MAKE: major adverse kidney events, eGFR: estimated glomerular filtration, ESKD: end stage kidney disease, CVD: cardiovascular disease.

*Hazard ratio is presented for All-cause mortality.

For CVD mortality as an outcome of interest, death due to any other cause is considered as competing event. For 50% GFR decline, ESRD and MAKE as outcomes of interest, non-renal death is considered as competing event. Hence, the sub-hazard ratios (SHR) reported for these outcomes.

Table 7: Risk of ESKD associated with the prescription usage and subject characteristics in the ICKD cohort.

	Unadjusted sub-hazard ratio (95% CI)	P value	Adjusted sub-hazard ratio (95% CI)	P value
**ACEI/ARBs**				
No®				
Yes	0.73 (0.60, 0.89)	<0.01	0.72 (0.58, 0.90)	<0.01
**Diuretics**				
No®				
Yes	1.24 (1.01, 1.52)	0.04	1.24 (0.99, 1.56)	0.07
**Calcium Channel Blockers**				
No®				
Yes	1.31 (1.08, 1.59)	0.01	1.16 (0.92, 1.48)	0.21
**Beta blockers**				
No®				
Yes	0.94 (0.76, 1.16)	0.58	0.90 (0.69, 1.16)	0.41
**Alpha blockers**				
No®				
Yes	1.04 (0.79, 1.36)	0.79	1.24 (0.92, 1.68)	0.15
**Central sympatholytic**				
No®				
Yes	1.11 (0.69, 1.79)	0.67	1.14 (0.65, 2.02)	0.65
**Aspirin**				
No®				
Yes	0.87 (0.69, 1.11)	0.27	0.90 (0.67, 1.21)	0.49
**Statin**				
No®				
Yes	0.69 (0.56, 0.85)	<0.01	0.65 (0.51, 0.83)	0.00
**Age**				
<60®				
60&above	0.80 (0.63, 1.01)	0.07	0.80 (0.61, 1.05)	0.11
**Sex**				
Female®				
Male	0.86 (0.71, 1.04)	0.12	1.06 (0.83, 1.35)	0.63
**Residence**				
Rural®				
Urban	0.77 (0.63, 0.94)	0.01	0.81 (0.63, 1.03)	0.08
**Income quartile (USD annual)**				
Q1: 0-1008®				
Q2: 1176-1680	0.97 (0.73, 1.29)	0.85	1.18 (0.85, 1.65)	0.32
Q3: 1764-4200	0.81 (0.62, 1.05)	0.12	0.96 (0.70, 1.32)	0.80
Q4: 4368-84000	0.86 (0.67, 1.12)	0.26	1.06 (0.77, 1.45)	0.74
**Heart fail**				
No®				
Yes	1.47 (0.60, 3.60)	0.39	1.05 (0.31, 3.61)	0.93
**eGFR**	2.27 (1.81, 2.86)	<0.01	2.07 (1.55, 2.77)	0.00

>=30®<br><30				
**Albuminuria**	1.90 (1.55, 2.33)	<0.01	1.91 (1.50, 2.42)	0.00
**Diabetes**<br>No®<br>Yes	 <br>1.29 (1.06, 1.56)	 <br>0.01	 <br>1.32 (1.05, 1.67)	 <br>0.02
**Obesity**<br>No®<br>Yes	 <br>0.74 (0.54, 1.00)	 <br>0.05	 <br>0.74 (0.52, 1.06)	 <br>0.10
**Stroke**<br>No®<br>Yes	 <br>0.75 (0.56, 1.01)	 <br>0.06	 <br>0.77 (0.56, 1.05)	 <br>0.10
**Systolic BP**<br><140®<br>>=140	 <br>1.27 (1.05, 1.54)	 <br>0.01	 <br>1.04 (0.83, 1.31)	 <br>0.71

MAKE: major adverse kidney events, eGFR: estimated glomerular filtration, ESKD: end stage kidney disease, CVD: cardiovascular disease.

*Hazard ratio is presented for All-cause mortality.

For CVD mortality as an outcome of interest, death due to any other cause is considered as competing event. For 50% GFR decline, ESRD and MAKE as outcomes of interest, non-renal death is considered as competing event. Hence, the sub-hazard ratios (SHR) reported for these outcomes.

Table 8 : Risk of CVD mortality associated with the prescription usage and subject characteristics in the ICKD cohort.

	Unadjusted sub-hazard ratio (95% CI)	P value	Adjusted sub-hazard ratio (95% CI)	P value
**ACEI/ARBs**				
No®				
Yes	0.95 (0.65, 1.39)	0.78	0.55 (0.34, 0.88)	0.01
**Diuretics**				
No®				
Yes	2.79 (1.88, 4.15)	<0.01	1.73 (1.09, 2.73)	0.02
**Calcium Channel Blockers**				
No®				
Yes	1.17 (0.79, 1.74)	0.44	1.08 (0.69, 1.69)	0.75
**Beta blockers**				
No®				
Yes	0.95 (0.61, 1.47)	0.82	1.05 (0.64, 1.72)	0.84
**Alpha blockers**				
No®				
Yes	0.90 (0.50, 1.63)	0.73	1.05 (0.56, 1.96)	0.88
**Central sympatholytic**				
No®				
Yes	0.51 (0.13, 2.06)	0.34	0.51 (0.16, 1.65)	0.26
**Aspirin**				
No®				
Yes	4.57 (3.08, 6.79)	<0.01	2.20 (1.35, 3.59)	<0.01
**Statin**				
No®				
Yes	3.79 (2.43, 5.90)	<0.01	2.01 (1.13, 3.57)	0.02
**Age**				
<60®				
60&above	1.47 (0.97, 2.25)	0.07	1.12 (0.68, 1.83)	0.66
**Sex**				
Female®				
Male	1.59 (1.01, 2.52)	0.05	1.67 (1.04, 2.69)	0.04
**Residence**				
Rural®				
Urban	0.55 (0.35, 0.87)	0.01	0.79 (0.48, 1.30)	0.35
**Income quartile (USD annual)**				
Q1: 0-1008®				
Q2: 1176-1680	0.82 (0.51, 1.32)	0.42	1.0 (0.57, 1.76)	0.99
Q3: 1764-4200	0.45 (0.27, 0.75)	<0.01	0.60 (0.34, 1.08)	0.09
Q4: 4368-84000	0.14 (0.06, 0.29)	<0.01	0.17 (0.07, 0.42)	<0.01
**Heart fail**				
No®				
Yes	16.09 (8.06, 32.12)	<0.01	11.70 (5.27, 25.98)	<0.01
**eGFR**	1.53 (0.88, 2.66)	0.13	1.67 (0.88, 3.19)	0.12

>=30®<br><30				
**Albuminuria**	2.17 (1.45, 3.24)	<0.01	2.14 (1.37, 3.34)	<0.01
**Diabetes**<br>No®<br>Yes	6.49 (4.07, 10.36)	<0.01	3.74 (2.09, 6.68)	<0.01
**Obesity**<br>No®<br>Yes	0.40 (0.17, 0.90)	0.03	0.39 (0.17, 0.91)	0.03
**Stroke**<br>No®<br>Yes	1.20 (0.72, 1.10)	0.48	1.36 (0.78, 2.35)	0.28
**Systolic BP**<br><140®<br>>=140	1.43 (0.96, 2.12)	0.08	0.74 (0.47, 1.17)	0.20

MAKE: major adverse kidney events, eGFR: estimated glomerular filtration, ESKD: end stage kidney disease, CVD: cardiovascular disease.

*Hazard ratio is presented for All-cause mortality.

For CVD mortality as an outcome of interest, death due to any other cause is considered as competing event. For 50% GFR decline, ESRD and MAKE as outcomes of interest, non-renal death is considered as competing event. Hence, the sub-hazard ratios (SHR) reported for these outcomes.

Table 9: Risk of all-cause mortality associated with the prescription usage and subject characteristics in the ICKD cohort.

	Unadjusted Hazard Ratio (95% CI)	P value	Adjusted Hazard Ratio (95% CI)	P value
**ACEI/ARBs**				
No®				
Yes	0.96 (0.77, 1.21)	0.76	0.77 (0.59, 1.00)	0.05
**Diuretics**				
No®				
Yes	2.22 (1.77, 2.79)	<0.01	1.95 (1.50, 2.53)	<0.01
**Calcium Channel Blockers**				
No®				
Yes	0.98 (0.78, 1.23)	0.85	0.90 (0.69, 1.18)	0.46
**Beta blockers**				
No®				
Yes	0.84 (0.65, 1.09)	0.19	0.90 (0.67, 1.21)	0.48
**Alpha blockers**				
No®				
Yes	0.84 (0.59, 1.20)	0.34	0.80 (0.53, 1.22)	0.30
**Central sympatholytic**				
No®				
Yes	0.66 (0.33, 1.33)	0.24	0.57 (0.23, 1.39)	0.21
**Aspirin**				
No®				
Yes	2.07 (1.63, 2.64)	<0.01	1.33 (0.99, 1.80)	0.06
**Statin**				
No®				
Yes	1.41 (1.12, 1.78)	<0.01	0.86 (0.65, 1.14)	0.29
**Age**				
<60®				
60&above	1.49 (1.17, 1.90)	<0.01	1.17 (0.89, 1.55)	0.26
**Sex**				
Female®				
Male	1.01 (0.80, 1.28)	0.93	1.10 (0.83, 1.46)	0.49
**Residence**				
Rural®				
Urban	0.71 (0.55, 0.90)	0.01	0.88 (0.66, 1.17)	0.38
**Income quartile (USD annual)**				
Q1: 0-1008®				
Q2: 1176-1680	1.04 (0.77, 1.39)	0.82	1.23 (0.88, 1.72)	0.23
Q3: 1764-4200	0.77 (0.58, 1.03)	0.08	0.89 (0.64, 1.25)	0.51
Q4: 4368-84000	0.33 (0.23, 0.47)	<0.01	0.37 (0.24, 0.56)	<0.01
**Heart fail**				
No®				
Yes	5.33 (2.89, 9.80)	<0.01	3.97 (1.99, 7.90)	<0.01
**eGFR**	1.59 (1.17, 2.16)	<0.01	1.46 (1.01, 2.11)	0.04

>=30®<br><30				
**Albuminuria**	1.74 (1.36, 2.22)	<0.01	1.54 (1.17, 2.04)	<0.01
**Diabetes**<br>No®<br>Yes	 <br>3.79 (2.99, 4.81)	 <br><0.01	 <br>2.85 (2.14, 3.79)	 <br><0.01
**Obesity**<br>No®<br>Yes	 <br>0.90 (0.64, 1.26)	 <br>0.53	 <br>0.85 (0.57, 1.25)	 <br>0.41
**Stroke**<br>No®<br>Yes	 <br>1.22 (0.90, 1.64)	 <br>0.19	 <br>1.32 (0.96, 1.81)	 <br>0.08
**Systolic BP**<br><140®<br>>=140	 <br>1.48 (1.18, 1.86)	 <br><0.01	 <br>1.03 (0.79, 1.34)	 <br>0.83

MAKE: major adverse kidney events, eGFR: estimated glomerular filtration, ESKD: end stage kidney disease, CVD: cardiovascular disease.

*Hazard ratio is presented for All-cause mortality.

For CVD mortality as an outcome of interest, death due to any other cause is considered as competing event. For 50% GFR decline, ESRD and MAKE as outcomes of interest, non-renal death is considered as competing event. Hence, the sub-hazard ratios (SHR) reported for these outcomes.
